# Case report of unusual insertion of the fibularis brevis muscle

**DOI:** 10.1007/s00276-022-02894-y

**Published:** 2022-02-22

**Authors:** Kacper Ruzik, Karolina Westrych, Marko Konschake, R. Shane Tubbs, Piotr Karauda, Łukasz Olewnik

**Affiliations:** 1 Department of Anatomical Dissection and Donation, Medical University of Lodz, Lodz, Poland; 2 Division of Clinical and Functional Anatomy, Department of Anatomy, Histology and Embryology, Medical University of Innsbruck, Müllerstr. 59, 6020 Innsbruck, Austria; 3 Department of Neurosurgery, Tulane University School of Medicine, New Orleans, LA USA; 4 Department of Neurosurgery and Ochsner Neuroscience Institute, Ochsner Health System, New Orleans, LA USA; 5 Department of Anatomical Sciences, St. George’s University, True Blue, Grenada; 6 Department of Neurology, Tulane University School of Medicine, New Orleans, LA USA; 7 Department of Structural and Cellular Biology, Tulane University School of Medicine, New Orleans, LA USA; 8 Department of Surgery, Tulane University School of Medicine, New Orleans, LA USA

**Keywords:** Anatomy, Ankle joint, Anatomical variations, Fibularis brevis, Fibularis digiti quinti

## Abstract

The fibularis brevis and fibularis longus muscles belong to the lateral compartment of the leg. The fibularis brevis is morphologically variable, especially in the number of tendons and place of insertion. Its type of insertion is correlated with the presence of a fibularis digiti quinti, which is also anatomically variable. We present a case study based on dissection of a seventy-three-year-old female cadaver with an unusual insertion of the fibularis brevis muscle. The tendon had three bands inserting into the fifth metatarsal bone. There was a coexisting fibularis digiti quinti, which was fused with the fibularis tertius muscle. Awareness of such anatomical variation could be useful during reconstructive surgery and planning rehabilitation protocols.

## Introduction

The fibularis brevis (FBM) is a slender muscle in the lateral compartment of the lower leg. This compartment also contains the fibularis longus muscle (FLM), which partly covers the underlying fibularis brevis [15, 16]. These clinically important bipennate muscles are innervated by branches arising from the superficial fibular nerve. They are supplied by the anterior tibial and fibular arteries and their primary function is foot eversion [1].

The FBM originates on the lower two-thirds of the lateral surface of the body of the fibula, medial to the FLM, and from the intermuscular septa that separates it from the adjacent muscles in the anterior and posterior compartments of the leg [13]. The fibers of the muscle belly merge into the fibularis brevis tendon (FBT). This is relatively flat and runs directly posterior to the distal fibula with the fibularis longus tendon (FLT) inside a common synovial sheath within a canal covered by the superior and inferior fibular retinacula [13].

According to anatomy textbooks, the FBM inserts into the tuberosity at the base of the fifth metatarsal bone on its lateral side. Recently, Olewnik et al. proposed a new twofold classification of FBM insertion in adults (types 1 and 2) [16].The FBM is important for the motor functions of the foot, assisting in its flexion and also in eversion of the inner portion of the foot. Each of these movements helps to keep the body balanced during walking on uneven surfaces [8].

The fibularis muscles are highly morphologically variable, with additional bands (fibularis brevis muscle and FLM), additional tendons, and additional muscles such as the fibularis digiti quinti (FDQ), fibularis tertius and fibularis quartus [4, 15, 16]. The present case report describes a very rare FBM inserting as three distinct bands and reveals a new variant of the course of the FBM tendon coexisting with the fibularis digiti quinti.

Knowledge of the morphological variability of this muscle is essential for orthopedic surgeons and physiotherapists. It is important to be aware of the anatomical makeup of the lateral leg compartment to understand potential pathologies and their implications for the function of the lower extremity.

## Case report

A female cadaver 73 years old at death was subjected to routine anatomical dissection for research and teaching at the Department of Anatomical Dissection and Donation, Anatomy and Histology, Medical University of Lodz. The left lower limb was dissected using standard techniques following a specified protocol [11, 15, 16].

Dissection began with removal of the skin and superficial fascia from the lateral compartment of the leg. Subcutaneous tissue and fascia were then gently removed to visualize the insertion of the FBM tendon and any additional bands. A FBM with an unusual insertion was observed. This FBM was photographed and subjected to further measurement using an electronic caliper with an accuracy of up to 0.1 mm (Mitutoyo Corporation, Kawasaki-shi, Kanagawa, Japan), each measurement being performed twice by two researchers. Consent to perform the anatomical stage was obtained from the Local Bioethical Commission (agreement no. RNN/297/17/KE).

**Fig. 1:**
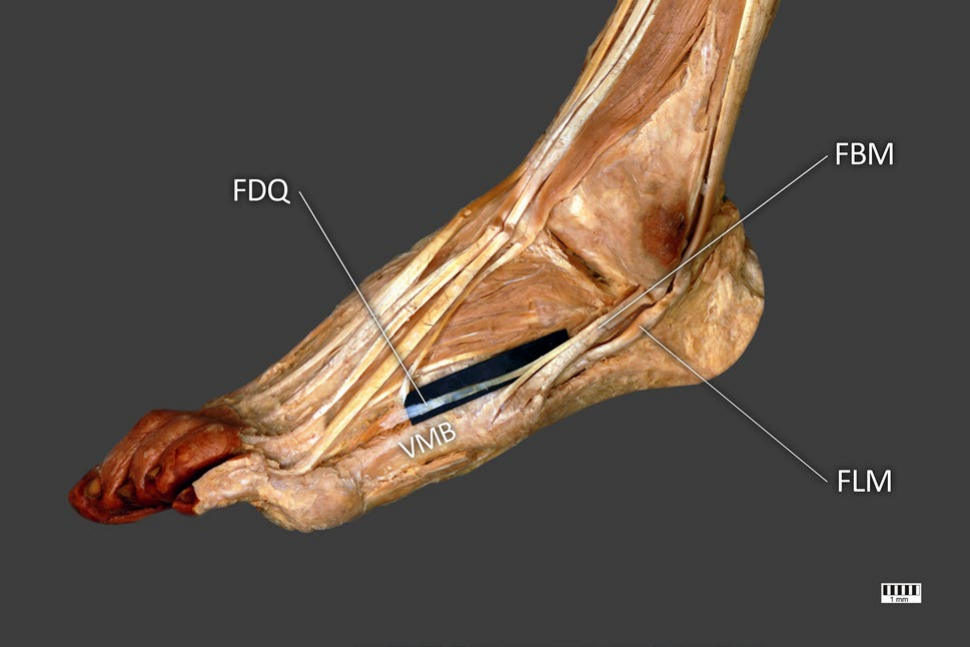
Case report of unusual insertion of Fibularis brevis. *FBM* fibularis brevis muscle, *FLM* fibularis longus muscle, *FDQ* fibularis digiti quinti, *VMB* V metatarsal bone

**Fig. 2:**
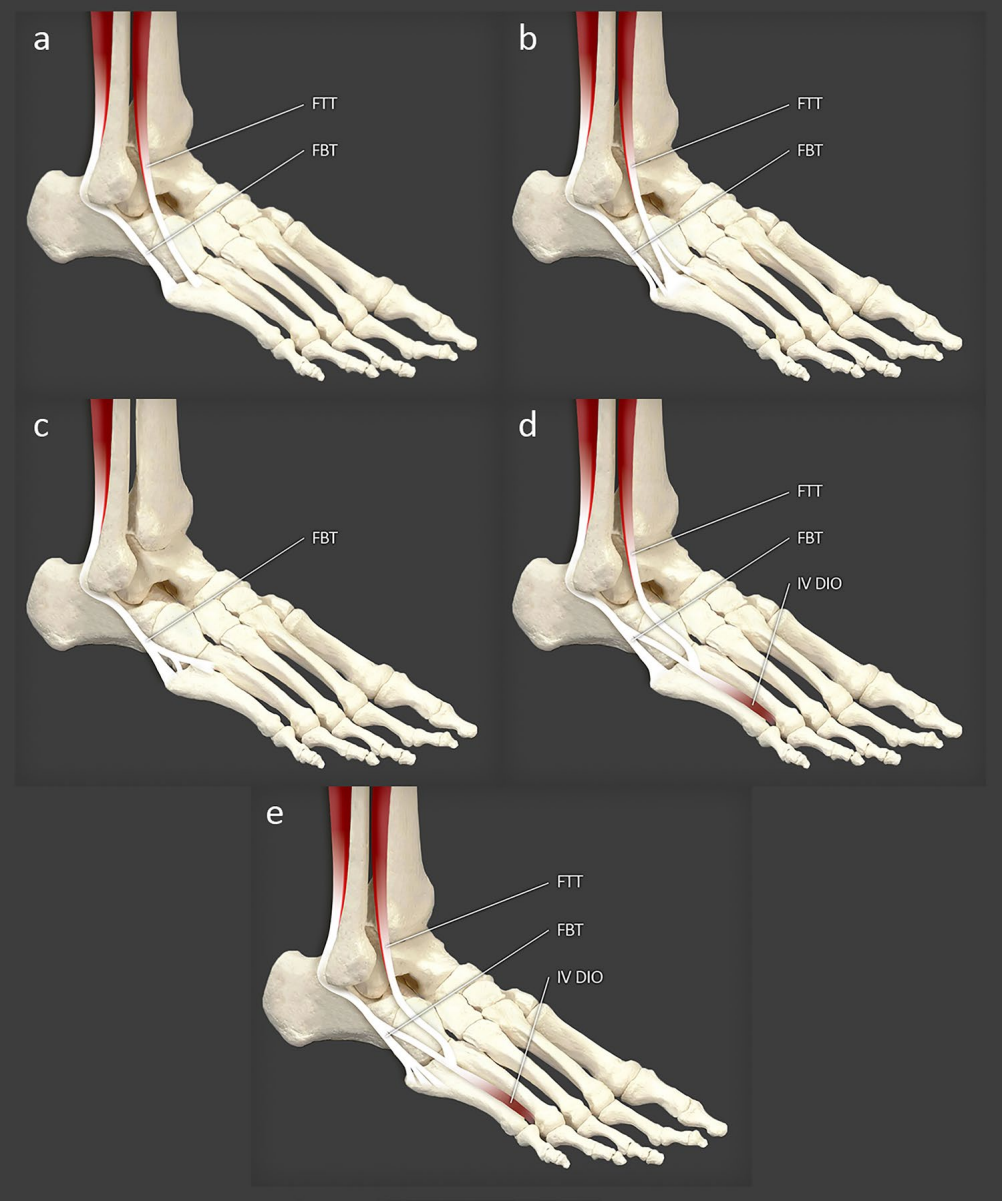
Types of FBM muscle according to Olewnik et al. **a** type 1, **b** type 2a, **c** type 2b, **d** type 2c, **e** our case report

The length of the muscle belly was 126.58 mm. Its width and thickness were 7.67 mm × 2.70 mm upon passing the muscle belly. After 54.75 mm, the tendon split into three terminating bands (Figs. 1, 2). This could correspond to type 2b in the Olewnik et al. classification [16]. However, an accessory band originated from the anterior band and was identified as the fibularis digiti quinti, which fused with the fibularis tertius, giving rise to the fourth dorsalis interosseus muscle. That connection was observed in the aforementioned classification but it always correlated with a type 1 FBM. (Figs. 1, 2) Table 1 gives the measurements of all the bands.

**Table 1:** Measurements of the fibularis brevis tendon

	Posterior band	Middle band	Anterior band	Fibularis digiti quinti
Width at the origin	2.12 mm	2.40 mm	1.68 mm	1.85 mm
Thickness at the origin	1.80 mm			1.42 mm
Length of the band	15.68 mm	20.57 mm	29.21 mm	51.88 mm
Width at the insertion	2.20 mm	2.32 mm	2.42 mm	2.10 mm
Thickness at the insertion	1.12 mm	1.25 mm	0.48 mm	1.12 mm
Location of insertion	Dorsal surface of the base of the fifth metatarsal bone	Dorsal surface of the base of the fifth metatarsal bone	Shaft of the fifth metatarsal bone	Fusion with the fibularis tertius

## Discussion

The types of origin and insertion have both been described in the literature. Macalister et al. described a slip band of origin to the abductor digiti minimi and another slip band joining to the fibularis longus tendon as an insertion variant, and two fibularis united in a deformed limb found by Ringhoffer [12, 18]. Cruveilhier reported an insertion of the FBM tendon to the tuberosity of the fifth metatarsal, and sometimes to a fibrous expansion to the 4th metatarsal [6]. Meckel divided the insertion of the FBM into shorter part, which attaches to the tuberosity of fifth metatarsal bone, and the longer part, which was subdivided into three parts. The following parts of the longer attachment were located on the middle part of the upper surface of the body of this bone, on the outer edge of the fourth tendon of the long common extensor of the toes, and on the posterior side of the fourth interosseous muscle. Humphry reported an insertion located on the outer surface of the fifth metatarsal bone and proximal phalanges and a thin tendinous slip passing forwards from it to join the extensor tendon [9]. Bardeen et al*. *described an extension to the tendon of the fourth and fifth toes and the fourth metatarsal bone [2]. Verma et al*.* described variants of insertion of the FBM to the tuberosity of the fifth metatarsal and the body of the fifth metatarsal bone [21]. Barghava et al*.* described possibilities for distal attachment of the tendon to the FBM; a slip tendon inserted to the head of fifth metatarsal bone, cuboid bone, calcaneus bone, fourth metatarsal bone, posterior tibiofibular ligament or middle and distal phalanx of the little toe [3]. Wood announced that the FTM sent a slip tendon to the extensor aponeurosis on both sides of the fifth toe [22]. LeDouble noticed a fibrous cord from the fibularis brevis tendon and described eight types of insertion of that structure [7]. He did not report fusion between that structure and any other muscle except the abductor of the fifth toe. He also reported a fibularis digiti quinti in36 limbs among a series of 100 subjects [7]. Interestingly, Chudzinski found it in half of all black subjects [5]. Testut described duplication of the fibularis brevis tendon. An additional tendon could insert into the fifth toe, or the abductor of the little toe, the fifth metatarsal, or on the fourth metatarsal and the fourth interosseous space [20].

Musiał described four types of possible distal attachment. The most common is insertion into the tuberosity of the fifth metatarsal bone [14]. The second most common has an additional band, which is inserted into the dorsal fascia of the foot at the level of the fourth interosseous space of the metatarsus. The third is characterized by an additional band inserted into the extensor aponeurosis of the fifth toe. The rarest type is trifurcated, i.e., inserted on the tuberosity of the fifth metatarsal bone, the extensor aponeurosis of the fifth toe and the dorsal fascia of the foot at the height of the fourth interosseous space of the metatarsus[14].

A fuller and more recent classification was proposed by Olewnik et al. [16]. It comprised two main types (I–II), Type II being further divided into sub-types. Type I (70.6%) was characterized by a single distal attachment in which the tendon inserts into the tuberosity at the base of the fifth metatarsal bone. Type II (29.4%) was characterized by a bifurcated distal attachment. The main tendon inserts into the tuberosity at the base of the fifth metatarsal bone on its lateral side. Three sub-types (A–C) were distinguished according to the site of attachment of the accessory slips: A—the accessory band inserts on the dorsal surface of the base of the fifth metatarsal; at the attachment point, the FBT connects to part of the fibularis tertius tendon. B—the accessory band splits into medial and lateral bands. The lateral band inserts to the dorsal surface of the base of the fifth metatarsal bone, while the stronger medial band inserts to the middle part of the body of the fifth metatarsal bone. C—the accessory band splits into medial and lateral bands. The lateral band inserts to the dorsal surface of the base of the fifth metatarsal bone, while the medial band fuses with the tertius fibularis, giving rise to the fourth interosseus dorsalis muscle [16].

In the present case, we found a Type II (according to the aforementioned classification) coexisting with a fibularis digiti quinti. This has not previously been reported.

The primary function of the fibularis longus and fibularis brevis is to provide the eversion moment necessary to balance the opposing inversion moments [17]. Several authors have reported the superiority of the fibularis brevis over the fibularis longus in the ability to evert and abduct the foot [17, 19]. These muscles are major evertors and are involved in complex actions such as dancing and skating. Experimental data support the hypothesis that the fibularis brevis tendon mechanism is more effective than the fibularis longus mechanism in rotating the navicular laterally and the calcaneus into the valgus; thus, fibularis tendons can be harvested for use in such procedures as tendon transfer and ligament reconstruction. This has clinical implications for assisting surgeons in selecting the tendon to harvest. Surgeons often deal with the loss of, or the need to sacrifice, one of those tendons [17].

An FDQ fused with in the fibularis tertius muscle could affect posture and the function of the lateral foot. Such a connection is antagonistic to the flexor digitorum brevis muscle as an additional tendon can also lead to clinical symptoms related to compression of adjacent vessels, nerves and tendons [4].

## Conclusion

Precise knowledge of the fibularis brevis muscle and its morphological variants is important because, on the one hand, complications resulting from additional bands of the FBM can manifest as persistent lateral ankle pain, and on the other, the FBM can be used as a graft in reconstructive surgery. It is also important for the surgeon to be aware of the multiple insertional variants; the fibularis can be injured after local hydrocortisone injection, or inadvertently during surgical procedures. More and better specific protocols for rehabilitation of patients with injuries to these muscles or their innervations could be developed in the future.

## Data Availability

Please contact authors for data requests (KacperRuzik—email address: kacper.ruzik@umed.lodz.pl).
